# De-diagnosis and the problem of revisability in adult ADHD and autism

**DOI:** 10.3389/fsoc.2026.1944117

**Published:** 2026-10-09

**Authors:** Marios Adamou, Darren Hill

**Affiliations:** 1 School of Human and Health Sciences, University of Huddersfield, Huddersfield, United Kingdom; 2 School of Health, Leeds Beckett University, Leeds, United Kingdom

**Keywords:** ADHD, autism, de-diagnosis, diagnosis, epistemic injustice, medicalisation, neurodiversity, sociology of diagnosis

## Abstract

Recorded diagnoses of attention-deficit/hyperactivity disorder (ADHD) and autism in adults have risen sharply across high-income health systems, and demand for assessment has risen faster still, while the best available population estimates of prevalence appear to have moved comparatively little. This paper treats that divergence as an analytic problem. We argue that adult neurodevelopmental diagnosis has undergone a threefold transformation: it has become demand-led and co-produced rather than professionally imposed, identity-conferring rather than merely descriptive, and self-reinforcing rather than static. The result is a diagnostic culture efficient at conferring diagnoses but weak, unsystematic and largely untheorised in reconsidering them. Drawing on the sociology of diagnosis, labelling theory, Ian Hacking’s account of interactive kinds and discourse-analytic studies of diagnostic contestation, we examine how demand-led medicalisation, the reframing of diagnosis as valued identity and the expansive use of the camouflaging construct can impede revision in practice. Against this background we develop the concept of diagnostic revisability: the capacity of a diagnostic culture to reconsider a diagnostic judgement, in either direction, in the light of change in the person or in the evidence. We distinguish correction of an insecure original judgement, revision in response to change, supersession and reversal of a non-diagnosis, and argue that revisability is an intrinsic property of diagnosis understood as defeasible inference. Precedents exist in medication review and deprescribing and in diagnostic de-labelling elsewhere in medicine. The case is stronger for ADHD, where symptoms and diagnostic status fluctuate and diagnosis is linked to prescribing, than for autism, where it rests principally on the security of the original assessment. Recent Swedish proposals for de-diagnosis services make revisability visible as an object of policy, but the conceptual case does not itself entail support for any service model. Diagnostic revision may correct error; it may also threaten identity, access to support and self-understanding, fall unequally on particular groups, or become a mechanism for rationing. If revisability is translated into practice, it must therefore satisfy stringent ethical and institutional safeguards, including an explicit equity criterion. The design requirements presented are minimum conditions for responsible revision and criteria for when it should not proceed.

## 1 Introduction

In 2025, health authorities in the Skåne region of Sweden began considering an innovation with no established institutional place in contemporary psychiatric practice: a formal service for reassessing, and where warranted removing, autism and ADHD diagnoses at the request of adults who no longer wish to carry them (Bowie, 2025). The proposal followed a pilot reassessment programme involving a small, self-selected group of people, most aged under 25, who had requested reassessment; most were found no longer to meet the diagnostic thresholds they had previously met. In a qualitative study of that programme, the diagnoses were characterised as “sticky,” meaning durable attachments, often formed in childhood, bound up with identity and access to resources, and capable of persisting long after their clinical relevance had faded (Lindkvist et al., 2026). The group was too small and self-selecting to carry population-level weight; the proposal’s significance lies in the conceptual problem it exposes: that psychiatric diagnostic systems are well developed for conferring categories at scale but far less developed for revising or withdrawing them, especially in the neurodevelopmental field.

This paper examines the asymmetry at the centre of that practice. In England, NHS England monitoring data cited by the Government’s independent review show the number of children and young people awaiting an ADHD assessment in mental health services rising from around 21,000 in April 2019 to around 270,000 by December 2025 (Department of Health and Social Care, 2026), and NHS England’s management information records up to 959,662 open referrals of all ages that may be for an ADHD assessment in June 2026, with the caveat that submissions from independent providers since February 2025 make the series non-comparable with earlier months (NHS England, 2026a). In June 2026 there were 294,792 patients of all ages with an open referral for suspected autism, and 17,509 new referrals in that month (NHS England, 2026b). These figures capture demand rather than conferral, but the indicators that track conferral point the same way. In UK primary care records, recorded ADHD diagnoses and prescriptions rose between 2000 and 2018 in every age group except the youngest, with the largest relative increases among adults (McKechnie et al., 2023), and the incidence of recorded autism diagnosis rose over the same period, most steeply among females and adults (Russell et al., 2022). The independent review concluded that population surveys, which do not rely on referral or diagnosis, suggest that the underlying prevalence of ADHD symptoms and of autism in adults has been far more stable, while cautioning that these data have limitations and that stable prevalence can coexist with sharply rising diagnosis, referral and service demand (Department of Health and Social Care, 2026).

That inference still requires caution, because adult prevalence estimates remain wide, method-dependent and contested. Community surveys commonly place adult ADHD at around 2.5 to 3 per cent (Fayyad et al., 2007; Simon et al., 2009) and adult autism at around 1 per cent, with successive adult psychiatric morbidity surveys in England producing estimates between 0.7 and 1.0 per cent (Brugha et al., 2011; Department of Health and Social Care, 2026); a meta-regression of three decades of prevalence studies found no evidence of an increase in the proportion of children meeting ADHD criteria when standardised procedures were followed (Polanczyk et al., 2014). We therefore use the claim only in the limited sense needed here: that growth in recorded diagnosis and assessment demand appears to have outpaced any plausible increase in the number of people who genuinely meet criteria. Similar gaps have been described in other high-income systems (Conrad and Bergey, 2014; Pellicano and den Houting, 2022). A stable underlying prevalence is compatible with rapidly rising recorded diagnosis for many reasons, and the divergence cannot by itself establish which of them is responsible. Historical under-recognition and the accumulation of previously undiagnosed adults, better recognition of presentations that do not match the childhood male prototype, reduced stigma, changed help-seeking, diagnostic substitution and new referral routes all predict growth in recorded diagnosis without any loosening of thresholds (Abdelnour et al., 2022; Russell et al., 2022); the competing account holds that thresholds have loosened and that ordinary variation is increasingly pathologised (Beeker et al., 2021; Honkasilta and Koutsoklenis, 2022; Kazda et al., 2021); the independent review concluded that several processes are likely to be operating at once (Department of Health and Social Care, 2026). We do not claim that the divergence settles the question, and nothing that follows depends on its being settled. Whatever the mix of mechanisms, diagnoses are now conferred at a scale and through routes that make it certain that some proportion of them, for reasons that differ between the two conditions, will need to be reconsidered, and the diagnostic culture has little capacity for doing so. Both explanatory camps treat the numbers as evidence about the rate of diagnosis; we treat them as evidence about what diagnosis has become, and about what happens after it is conferred.

Our argument is that adult neurodevelopmental diagnosis has been transformed in three related ways. It has become increasingly demand-led and co-produced rather than professionally imposed, so that the pressure to diagnose now flows in substantial part from people seeking the label, in interaction with clinicians, advocacy organisations, healthcare markets and the administrative systems that attach entitlements to diagnosis. It has become identity-conferring, so that a diagnosis functions less as a description of impairment than as an account of the self and a claim to membership of a valued minority. And it has become self-reinforcing, so that the category expands and stabilises through the very processes that classify people under it. The joint effect is a diagnostic culture that is efficient at conferral and weak at reconsideration, and the Swedish proposals make that weakness visible and contestable.

We do not argue that diagnostic revision is technically impossible, nor that the system contains no mechanisms for it: an annual medication review is recommended for ADHD, a deprescribing consensus recognises misdiagnosis, diagnostic de-labelling is established in other fields of medicine, and second-opinion pathways exist. The claim is narrower: that these processes are unsystematic and lack a clear clinical pathway, so that the diagnostic culture often behaves as though revision were not one of its recognised functions. The distance between a mechanism that exists on paper and one that is recognised, resourced and routinely used is the space this paper examines.

We develop this as a conceptual analysis in the tradition of the sociology of diagnosis (Blaxter, 1978; Brown, 1995; Jutel, 2009; Jutel and Nettleton, 2011), illustrated at two points by findings previously reported in discourse-analytic studies of adults who contest assessments when a diagnosis is not made (Adamou et al., 2026a, 2026b). Those studies are used to show a mechanism, not to estimate its prevalence, and each mechanism is also grounded in literature independent of the authors’ own programme: qualitative research on diagnosis-seeking and late diagnosis (Hansson Halleröd et al., 2015; Leedham et al., 2020; Lilley et al., 2022; Young et al., 2008), the sociology of labelling and stigma, the overdiagnosis and de-labelling literatures, and the compensation and camouflaging research itself.

The paper first states the problem, then sets out a typology of diagnostic revision and defines revisability, then analyses the three mechanisms that foreclose revision in practice, before showing that a professional infrastructure for revision already exists, treating the ethics and politics of diagnostic removal, and setting out what revisability would require. The theory generates testable predictions: that annual ADHD reviews will rarely result in diagnostic revision despite formally being an occasion at which continuing clinical need is reassessed, that contestation of non-confirmed assessments will be organised around identity rather than treatment in autism and around treatment in ADHD, and that expansive invocations of camouflaging will be more frequent where observed presentation diverges from claimed diagnosis. Each is open to examination in service records, discourse-analytic corpora and audit data.

The paper thus advances two distinct arguments. The first is conceptual and epistemic: the revisability of a diagnosis is a necessary property of any diagnostic culture that treats diagnosis as a provisional judgement rather than an irreversible fact, and this capacity is underdeveloped in adult neurodevelopmental psychiatry. The second is conditional and normative: if this capacity were translated into practice, its exercise would be legitimate only under stringent safeguards concerning initiation, independent scrutiny, financial influence, continued access to support, equity and the unequal power of patients and services. The design requirements in the penultimate section are not a specification for a de-diagnosis service but the minimum conditions for responsible revision and the criteria for determining when revision should not proceed; the conceptual case for revisability does not, by itself, commit the reader to any service model.

### 1.1 Positionality and the evidential basis of the argument

A paper that analyses professional power over identity should state where its authors stand. The first author is a consultant psychiatrist who leads an adult ADHD and autism service in the National Health Service in England; the second-opinion requests and complaints analysed in the studies drawn on here were addressed to services of that kind, and he is also the author of the critiques of camouflaging on which part of Section 6 builds. The second author is an academic in social work with a professional background in community social care and no clinical role in neurodevelopmental assessment. This positioning gives the analysis familiarity with the previously published findings and a practitioner’s view of how revision is foreclosed, but it carries risks: the first author belongs to the group whose decisions the contesting corpora dispute, and clinicians have interests in the allocation of diagnostic authority that patients and advocacy organisations do not share. We have tried to guard against these risks by stating the limits of our own studies wherever they are used, grounding each mechanism in independent literature, stating the strongest counter-positions before answering them, and treating the ethical constraints on revision as binding conditions rather than caveats. Readers should nevertheless weigh the argument with this positioning in view.

## 2 The problem: a sociology of naming and the under-development of diagnostic revision

The sociology of diagnosis begins from a claim that is now well established, but whose implications remain underdeveloped: that to diagnose is not simply to detect a pre-existing entity but to perform an act of classification which has social agency. Blaxter's (1978) foundational study of alcoholism treated diagnosis as simultaneously a category and a process, a stable label and an ongoing activity through which clinicians sort ambiguous presentations into recognised kinds. Brown (1995) distinguished the discovery of conditions from the framing that renders them diagnosable, treatable and administratively actionable. Jutel’s programmatic work consolidated the field, arguing that classification “shapes medicine and guides its practice”, that diagnosis is a boundary object linking the biological, the professional and the bureaucratic, and that it operates as a form of power, the authority to confer or withhold the sick role, and with it access, exculpation and identity (Jutel, 2009, 2011; Jutel and Nettleton, 2011).

This literature has been overwhelmingly concerned with how conditions become diagnoses and how diagnoses are conferred; its centre of gravity runs toward naming. Freidson's (1970) account of professional dominance describes medicine’s jurisdiction as the authority to define what counts as illness, a power exercised in the act of conferral. The reverse operation has not gone wholly unexamined. Sociology has a real, if smaller, literature on demedicalisation: Conrad and Schneider (1980) traced how conditions such as masturbation and, most consequentially, homosexuality were removed from the medical domain, and Conrad (2007) later took the shifting status of such conditions as evidence that medicalisation is not a one-way ratchet. What that literature almost never addresses is revision at the level of the individual case within a live clinical relationship, the withdrawal of a particular person’s diagnosis while the category itself remains, and it is virtually silent on this operation in adult neurodevelopmental psychiatry. That is the gap this paper addresses.

Sociology also has a classical account of why labels stick, and it bears directly on the “stickiness” the Swedish participants described. Labelling theory held that a psychiatric label, once applied, is stabilised by the responses of others and by the labelled person’s adoption of the role (Scheff, 1966), and Goffman (1963) described the management of a spoiled identity as continuing work performed long after the labelling event. Modified labelling theory added that the labelled person’s internalised expectations of devaluation have consequences independent of the label’s accuracy (Link et al., 1989), and Link and Phelan (2001) defined stigma as the co-occurrence of labelling, stereotyping, separation, status loss and discrimination in a context of power. This tradition explains stickiness through stigma: the label persists because it is discrediting and because institutions act on it. The neurodevelopmental case both confirms and inverts that account: the label is acted on by institutions, but it is often sought rather than resisted, and its durability owes as much to its value as to its cost. Rosenberg's (2002) account of the “tyranny of diagnosis” explains why: once disease is conceived as a specific entity, diagnosis becomes the indispensable link between the person, clinical practice and bureaucratic institutions, and a diagnosis, once recorded, acquires an administrative life of its own. Eyal et al. (2010) showed that the expansion of autism diagnosis was produced by a shifting matrix of parents, clinicians, therapies and institutions rather than by any single actor, an account that anticipates the co-produced demand described in Section 4. Timmermans and Buchbinder (2010) described “patients-in-waiting,” held between health and disease; the present analysis concerns the reverse trajectory, people held within a category after its warrant has weakened, for whom no comparable liminal status exists.

The overdiagnosis literature comes closest to the problem addressed here. Moynihan et al. (2012) and Welch et al. (2011) described how people come to be diagnosed with conditions that will never harm them, Brodersen et al. (2018) distinguished overdetection from overdefinition, the widening of criteria to include milder presentations, and Hofmann (2016) argued that medicalisation and overdiagnosis are converging. Most of this work concerns how unwarranted diagnoses arise; it says less about what should happen to a diagnosis already conferred. The exception is Hofmann and Lea (2022), who examined the ethics of withholding and withdrawing diagnoses that do not satisfy professionally accepted criteria. They identified the moral functions that make such diagnoses hard to remove, including their explanatory role, their conferral of access to care and social rights, and their place in identity construction; they described psychological mechanisms in patients (the endowment effect, loss aversion, status quo bias) and in professionals (aversion asymmetry, anticipated regret, the imperative of action); and they concluded that, because withdrawal changes a status that withholding leaves untouched, it is more difficult to withdraw than to withhold a diagnosis. Their analysis, which mentions ADHD only in passing, supplies from outside the authors’ own research programme the asymmetry this paper examines. We build on it by specifying the sociological mechanisms that make the asymmetry unusually strong where the diagnosis is sought, identity-conferring and self-reinforcing; by distinguishing the withdrawal of an unwarranted diagnosis, which is their subject, from forms of revision they do not treat, including revision in response to change and reversal of a non-diagnosis; and by situating revision within the institutional architecture of entitlement and professional power that determines whether it can be done responsibly at all.

The same professional authority that Freidson identified as the power to name is, logically, a power to un-name. Yet the removal of diagnosis at the level of the case has little developed sociology, almost no professional protocol, and, as the Swedish proposal reveals, barely any institutional existence (Hofmann and Lea, 2022). We propose that this absence follows from a feature of the dominant frame itself. Because the sociology of diagnosis has been so concerned to demonstrate that diagnosis is constructed and consequential, it has tended to treat the conferred diagnosis as a settled social fact whose effects then unfold. The temporal structure of the diagnosis, its provisionality, its dependence on information available at a given moment, and its susceptibility to inaccuracy as the person or the evidence changes, has received less attention than its performative force. Reintroducing that temporal structure is the first move of this paper: a diagnosis is a formulation indexed to a time, its accuracy is not guaranteed to persist, and a diagnostic culture that cannot register this has mistaken a judgement for a fact. Revisability is therefore not a technical addition to diagnosis but a condition of its integrity as a form of knowledge.

## 3 Revisability and its forms

Before analysing why revision is foreclosed, it is necessary to say what revision is, because the term “de-diagnosis” has been used, in the Swedish debate and in our own earlier formulations, to cover processes that are epistemically and clinically distinct.

The starting point is the logical character of diagnosis itself. A clinical diagnosis is not a deduction from criteria but an abductive inference: an inference to the best available explanation of a pattern of symptoms, history and observation, provisional by construction and open to revision as evidence accumulates (Stanley and Campos, 2013). Diagnostic criteria discipline that inference; they do not replace it. On this understanding, revisability is not an optional addition to diagnosis but an intrinsic property of diagnostic reasoning, and a diagnostic culture that cannot register new evidence has stopped reasoning diagnostically. This does not make every diagnosis doubtful; it makes the warrant for a diagnosis a matter of the evidence available at a time, and both the evidence and the person can change.

At least five situations follow, and they call for different responses. The first is correction: the original judgement was not securely established, because the assessment was inadequate, the evidence was misread or the criteria were not in fact met. The diagnosis was unwarranted from the outset, in Hofmann and Lea's (2022) sense, and the response is to correct the record and explain why. The second is revision in response to change in the person: the original judgement was sound, but the person no longer meets criteria, or meets them only partially. The historical diagnosis remains accurate as a statement about the past, and the response is to record a change of current status rather than to delete the history. DSM-5 already provides for this in ADHD through the specifier “in partial remission”, applied when full criteria were previously met, fewer than the full criteria have been met for the past 6 months and the symptoms still result in impairment (American Psychiatric Association, 2013). The third is supersession: the presentation is better explained by a different formulation, so that the earlier diagnosis is replaced rather than removed. The fourth is administrative correction of a record that misstates what was concluded. The fifth, which the debate on de-diagnosis has almost entirely neglected, is reversal of a non-diagnosis: the reconsideration of a conclusion that criteria were not met, in the light of new evidence, a second opinion or a change in the person. A diagnostic culture that could revise diagnoses but not non-diagnoses would not be revisable; it would merely be restrictive, and it would deserve the suspicion that it served rationing rather than accuracy.

We therefore define the revisability of diagnosis as the capacity of a diagnostic culture to reconsider a diagnostic judgement, in either direction, in the light of change in the person or in the evidence, and to record the outcome in a form that distinguishes correction from remission and supersession. This is broader than the definition we offered previously, which was restricted to diagnoses “previously made responsibly”. That restriction was unstable, because much of the case for revision in autism concerns judgements whose security is precisely what is in doubt; the security of the original judgement is better treated as a ground for revision, the one that distinguishes correction from the other forms, than as a condition of eligibility for it. The term “de-diagnosis” is retained for the removal of a diagnosis from a person’s current clinical status, by correction, remission or supersession; it is one outcome of revisability, not a synonym for it.

The typology has a consequence for the analogy with deprescribing developed in Section 8. Medication is a current intervention whose indication can cease; when it does, the medication is stopped and the historical fact that it was once indicated is not disturbed. A diagnosis has both a current and a historical aspect, and the analogy holds only for the current aspect: whether the diagnosis continues to be affirmed, to authorise treatment and to carry entitlements. It does not license the rewriting of a historical judgement that was sound when made.

The typology also clarifies the existing asymmetry of the system. Adult neurodevelopmental services in England already possess a route by which a non-diagnosis can be contested and, in principle, reversed: the second-opinion request and the formal complaint, the very processes analysed in the corpora drawn on below. No equivalent route exists by which a conferred diagnosis is routinely reconsidered. The asymmetry this paper examines is therefore revision that runs in one direction only, towards conferral, and the reasons are not merely administrative. Three features of contemporary adult neurodevelopmental diagnosis actively foreclose it in practice: a change in the direction of demand; a change in what the diagnosis does for the person who holds it; and a construct that can insulate the diagnosis from disconfirmation.

## 4 The demand-led turn: medicalisation from below

The classical theory of medicalisation was, in its origins, a theory of imposition. Conrad’s body of work traced how domains of ordinary life, such as hyperactivity in children, sadness, shyness and ageing, were brought under medical jurisdiction, and located the engines of that expansion in professional interests, pharmaceutical markets and the cultural authority of biomedicine (Conrad, 2005). Szasz's (2007) polemic against the medicalisation of everyday life, whatever its excesses, shared the same directional assumption: that medicine, and the interests aligned with it, expand their reach over populations who are, in the main, the objects rather than the authors of that expansion (Montagne, 2008). In this frame, the patient is diagnosed; the verb is passive.

The contemporary position complicates this direction of influence without displacing the older forces. Conrad and Bergey (2014) had already noted the growing role of consumers, advocacy groups and direct-to-consumer information in the globalisation of ADHD, and Eyal et al. (2010) showed that the expansion of autism diagnosis was the work of an alliance of parents, clinicians and institutions rather than of professional imposition alone. Since then this trend has intensified, and in adult neurodevelopmental practice a substantial and growing part of the pressure to diagnose now appears to come from people actively seeking a diagnosis. This is a directional claim, not a quantified one, and it is a claim about co-production rather than about a simple reversal of direction. Contemporary medicalisation is produced jointly by patients, clinicians, advocacy organisations, independent providers operating in a healthcare market, and the administrative systems that attach entitlements to diagnosis. The older engines have not disappeared: NHS expenditure on private ADHD providers rose steeply between 2022 and 2025, and supply-side incentives remain active (Rowland, 2026). The available evidence shows that patient-led demand is real, organised and consequential, but it does not establish what proportion of total diagnostic pressure it represents, and no current data source can. Independent qualitative research does show that adults commonly arrive at assessment having already identified with the category and seeking confirmation of an explanation for a difficult life, and that the diagnosis, when made, is experienced as explaining a previously inexplicable history (Hansson Halleröd et al., 2015; Leedham et al., 2020; Lilley et al., 2022; Young et al., 2008). The claim is also visible in the growth of self-identification, the routing of demand through patient-choice mechanisms and independent providers, and, most clearly, the response that can follow when a diagnosis is not confirmed.

A critical discourse analysis of written requests for a second opinion, submitted to a National Health Service neurodevelopmental service by adults whose assessments had concluded that they did not meet diagnostic criteria, described the discursive work through which requesters contest the clinician’s conclusion (Adamou et al., 2026a). They appropriate and recalibrate clinical taxonomy, redeploying diagnostic vocabulary against the assessor; they marshal biographical and pharmacological evidence; they reframe the clinician’s benevolence as misunderstanding; and they invoke masking to reconcile a claimed condition with an unremarkable observed presentation. The analysis also names a structural bind, the competence paradox, in which the fluency needed to mount a persuasive challenge can itself be read as evidence against the impairment claimed. A companion rhetorical analysis of complaints that were not upheld describes a consistent structure in which a negative outcome is recast as service failure, medical trauma and procedural breach, with complainants typically demanding a specific outcome, most often a prescription or the confirmation of a particular diagnosis (Adamou et al., 2026b).

We do not treat these findings as evidence of prevalence, nor as judgements on the merits of individual cases. The requesters are, by definition, a specific and unrepresentative subgroup of people whose assessments ended in diagnostic non-confirmation and who then chose to contest that outcome. What the corpora can show is that patient-led contestation exists and how it is organised; what they cannot show is that adult neurodevelopmental diagnosis has in general become demand-led rather than professionally driven or jointly produced, and we do not claim that they do. Their value is that they make visible a reversal in the usual direction of medicalisation: the people in these corpora are not being medicalised against their will but are seeking medicalisation and experience its refusal as an injustice.

The classical critique assumed that the scarce and contested good was protection from diagnosis; here it is diagnosis itself. This is medicalisation from below, a shift others have registered in arguing that medicalisation is now achieved substantially through the efforts of patients and citizens seeking to legitimise their distress in medical terms (Correia, 2017), and it changes the political economy of the diagnostic encounter. When demand is professional or commercial, the corrective to over-diagnosis is to restrain the supplier; when demand is in substantial part patient-led and morally charged, restraint reads as denial, and the clinician who declines to diagnose is cast not as a steward of the category but as an obstacle to a rightful claim.

## 5 The self-reinforcing category: identity, neurodiversity and interactive kinds

Why has demand taken this form, and why does it feed on itself? The answer lies in what the diagnosis has come to do for the person who receives it. For a growing number of adults, and disproportionately for women who reached mid-life without an explanation for a lifetime of difficulty, a diagnosis of autism or ADHD arrives less as a clinical verdict than as a biography made suddenly legible: failures at school, exhaustion at work and friendships that never quite held acquire a cause and a name, and the name comes with a community of others who describe the same life. Where Bury (2001) described how people narrate an illness that has disrupted a settled life, the neurodevelopmental diagnosis often does the opposite work, supplying the missing thread that makes an already disrupted life cohere: the diagnosis is not the rupture in the story but the story’s explanation. Independent studies of late-diagnosed adults describe exactly this re-reading of a life through a new lens, and the relief, and sometimes the grief, that accompanies it (Leedham et al., 2020; Lilley et al., 2022).

This reorganisation is frequently accurate and beneficial. There is a genuine history of under-recognition, particularly among adults and among women and girls whose presentations did not match the male childhood prototypes on which the categories were built, and for these people a late diagnosis can end years of misattributed failure and unlock support that materially improves their lives. The point of what follows is not that the meaning people find in diagnosis is illusory, but that the same processes which make diagnosis so valuable to hold also make it structurally resistant to revision, and that these are separable questions.

What people expect of a diagnosis is not uniform. A thematic analysis of adults referred for autism assessment found that they hoped, above all, for personal growth and self-understanding, for an explanation they could offer to others, and for inclusion and acceptance, with access to practical support being secondary (Adamou et al., 2025). A parallel study of adults referred for ADHD assessment found a markedly different pattern, organised around legitimisation and practical remedy, with nearly half raising medication unprompted against almost none of the autism group (Adamou and Wharton, 2026). Many autism participants positioned themselves in advance as interpreters of their own difference to family, employers and clinicians: identity-work in the strict sense, in which the diagnosis is sought less to obtain a treatment than to authorise a self and a standing. Those who wrote to contest a negative assessment were likewise, for the most part, seeking not a treatment they had been denied but recognition of an identity they had already assumed; to be told they did not meet criteria was, in their own accounts, to have a self-refused (Adamou et al., 2026a).

We propose that two features of the present situation turn this personal meaning into a self-reinforcing social process. The first is that the formal diagnosis now sits at the entrance to a set of tangible goods. In the United Kingdom, the Equality Act 2010 defines disability by reference to a physical or mental impairment that has a substantial and long-term adverse effect on a person’s ability to carry out normal day-to-day activities (Equality Act, 2010, s. 6). A diagnosis is neither necessary nor sufficient for that definition to be met. In administrative practice, however, employers, educational institutions and adjudicators commonly treat a formal diagnosis as the evidential basis on which impairment is recognised and adjustments are agreed, so that the label operates as the practical means of access to workplace adjustment, examination access, educational support and, in some circumstances, welfare assessment. The gap between a functional legal test and a diagnostic administrative practice is itself part of the phenomenon this paper describes. Whatever the neurodiversity movement’s insistence that these are differences rather than deficits, their administrative recognition is mediated by a medical label. Here is the paradox at the centre of the movement’s politics: a paradigm whose main claim is that autism and ADHD are not disorders coexists with the demand for their medical diagnosis, and the clinical label has become the passport to a non-medical identity and to the accommodations that identity claims.

The second feature is self-identification, which now runs largely outside the clinic: autistic and ADHD identities circulate on social media as checklists, first-person accounts and community affiliation, and a person may inhabit the identity for years before seeking, or instead of seeking, a formal assessment. The neurodiversity movement supplies the scaffolding for this. It grew out of autistic self-advocacy in the early 1990s and was developed collectively within that community rather than coined by any single author (Botha et al., 2024; Pellicano and den Houting, 2022); in its stronger statements it treats neurominorities as oppressed groups whose disablement is produced by an intolerant environment rather than by any internal pathology (Walker, 2021; Chapman, 2023), a perspective carried into theoretical frameworks in neuroscience itself (Kokorikou et al., 2023). It would be a simplification to present the movement solely as an engine of demand, because the neurodiversity literature contains its own internal debates: about whether autism is best understood as deficit, difference or both (Kapp et al., 2013), about the relationship between self-identification and clinical diagnosis, and about the ways in which research and clinical framings construct autistic people (Botha and Cage, 2022). The dynamic we describe is therefore a property not of the movement’s ideology but of the administrative fusion of identity, diagnosis and entitlement within which it operates and which it often criticises; the effect, whatever the intentions of its theorists, is to make the identity worth claiming and defending, and to cast the clinician who withholds its formal confirmation as an obstacle to a recognised good.

Ian Hacking’s work on interactive kinds supplies the mechanism (Hacking, 1986, 1999, 2007). The classifications of the human sciences differ from those of the natural sciences in one decisive respect: the people classified know that they are classified, and they respond, adopting the category, modelling themselves on it, resisting or elaborating it, and in doing so changing what the category picks out. The kind and its members move together; Hacking called this the looping effect and described human kinds as moving targets. Adult neurodevelopmental diagnosis has become a textbook case. The category is engineered and re-engineered through successive revisions of the diagnostic manuals, through the instruments that operationalise it, and through the self-descriptions of those who claim it, and each revision loops back onto a population that is itself shifting in response.

Two things must be kept apart here. The first is what Hacking’s framework can establish: that a classification which people take up alters self-description, help-seeking and collective identity, and that the category and its members therefore move together. That is sufficient to explain why self-identification precedes referral and referral yields recognition, why attachment runs deep, and why removal of a diagnosis can be experienced as erasure. The second is what the framework cannot establish: that the category has expanded beyond its valid boundaries, that increasing recognition amounts to dilution, or that growing heterogeneity entails declining validity. Those are claims about clinical accuracy, and they require independent evidence. The widening of the category beyond the severe, early-onset prototypes of the founding studies may reflect the recognition of people whom earlier criteria missed or the inclusion of presentations that do not meet criteria; the looping effect is consistent with both and cannot decide between them. We therefore use Hacking only for the first set of claims, and the consequence for revisability follows from them alone: as the category expands and its members invest in it, the diagnosis hardens into a way of being, and the same looping that drives the category outward makes the removal of a diagnosis feel like erasure, whatever the accuracy of any particular diagnosis.

The literature on biological citizenship describes the same dynamic: a biomedical category becomes the basis for collective identity, claims on the state and a shared moral community, so that scepticism about the category is experienced not as a clinical disagreement but as an attack on the community formed around it (Petryna, 2002; Rose and Novas, 2005). That literature treats identity formation as a rational response to a real institutional situation rather than as fashion, grievance or ideological excess, and we follow it. The population under discussion includes people with genuine conditions, real histories of discrimination and hard-won forms of self-understanding, and any adequate analysis has to take those realities seriously.

## 6 The resistance of revision: camouflaging and the insulation of diagnosis from disconfirmation

The third mechanism is conceptual and concerns the role that camouflaging, or masking, has come to play in adult neurodevelopmental diagnosis. The concept describes a real and clinically important phenomenon, the suppression, compensation or modulation of traits to meet social expectations, which helps explain why some people, particularly adults and women, were missed earlier in life despite genuine impairment. The difficulty is that the same concept can make diagnosis resistant to revision. When observable traits are present, they support the diagnosis; when they are absent, their absence may be explained as masking, and limited developmental evidence may be attributed to compensation, concealment or late recognition. Camouflaging can therefore shift from an explanation for missed cases to a means of insulating the diagnosis from disconfirmation, absorbing evidence and counter-evidence into the same account and narrowing the conditions under which revision can occur.

The critique that follows is directed at a particular inferential use of masking, not at the phenomenon itself, and it is important to begin by recognising the construct in its strongest and most legitimate form. Masking explains a real clinical problem, that some people, most notably late-diagnosed autistic women, learn to suppress, compensate for or work around their difficulties, and it has a plausible mechanism of sustained, effortful compensation, often exhausting and associated with later mental health costs (Milner et al., 2019). It also corrects a genuine historical bias in assessment, which has often been shaped by male and childhood presentations (Rosen et al., 2021). The possibility of masking is, moreover, built into the current criteria: DSM-5 states that the symptoms of autism must be present in the early developmental period but “may be masked by learned strategies in later life” (American Psychiatric Association, 2013). The critique is therefore not directed at the recognition of masking as such, which the criteria require, but at the way in which it is sometimes invoked. Used in this bounded, developmentally anchored way the construct is legitimate; it is sometimes used in a much stronger and more epistemically damaging form.

In that stronger deployment, which we observed in the second-opinion corpus described above and which we take to be a specific pattern in contested assessments rather than a description of assessment in general, masking is invoked to reconcile a claimed diagnosis with a current presentation that does not meet criteria, so that the very absence of observable features is taken as evidence of the condition, successfully concealed. Every negative observation can then be reinterpreted as successful masking, and no observation could, even in principle, count against the claim. The philosophy-of-science frame implicit here should be made explicit. Popper (1959) described an auxiliary hypothesis introduced solely to rescue a theory from refutation as a conventionalist stratagem that reduces the theory’s empirical content, and Lakatos (1970) distinguished progressive adjustments to a research programme, which predict new facts, from degenerating ones, which merely accommodate anomalies after the event. The stronger use of camouflaging has the marks of the degenerating case: it is invoked after the fact, makes no independent prediction, and grows in explanatory scope precisely as disconfirming evidence accumulates. Two qualifications limit the frame. An individual clinical formulation is not a scientific theory, and falsification is the wrong standard for it; the relevant standard is whether some observation could lower its probability, and a formulation to which no observation could make any difference has ceased to be a formulation. And because the criteria themselves acknowledge masking, the legitimate and the degenerating uses differ not in whether masking is invoked but in how: whether it is anchored in developmental history and corroboration, and whether it is treated as a hypothesis that could fail.

The construct’s difficulties have been set out more fully elsewhere (Adamou, 2026; Adamou and Koutsoklenis, 2026) and are summarised here only in the form relevant to revisability. First, the boundary of the construct is unstable. As commonly used, masking does not identify a class of phenomena that can be clearly separated from the ordinary human practice of adjusting self-presentation to context, and the compensatory strategies reported by autistic adults are qualitatively similar to those reported by adults with social difficulties and no diagnosis (Livingston et al., 2019). If everyone masks, the concept loses its discriminating power at the level of the individual case. Second, its measurement is theory-laden in a way that matters for diagnosis. Of the two operationalisations that dominate the literature (Cook et al., 2021), the discrepancy approach defines camouflaging as the gap between an “internal” status, measured by self-reported autistic traits and mentalising performance, and an “external” presentation, measured by clinician observation (Lai et al., 2017). Applied to an individual, this definition converts any excess of self-report over observation into evidence of camouflaging, when it could equally reflect over-reporting, measurement error or the absence of the condition; the discrepancy cannot distinguish these, because it is defined as camouflaging. The self-report approach, exemplified by the Camouflaging Autistic Traits Questionnaire, was developed from autistic adults’ accounts of camouflaging and codes practices such as monitoring one’s expressions, rehearsing social scripts and copying others’ mannerisms as camouflaging; its convergent validity was established against measures of autistic traits, anxiety and depression (Hull et al., 2019). A high score therefore cannot, on its own, discriminate a camouflaged neurodevelopmental condition from social anxiety or ordinary self-presentational effort, and the construct validity of both approaches has been debated within the camouflaging literature itself (Ai et al., 2022; Fombonne, 2020; Lai et al., 2021; Williams, 2022). The circularity we have in mind is thus specific: the instruments were built from the reports of people already identified with the category and validated against the traits they are meant to explain, so that an affirmative self-report of adaptive effort becomes evidence of the condition rather than a hypothesis about it. Third, the stronger use raises a question of evidential weight rather than of logical possibility. Compensation research treats camouflaging as effortful, learned and imperfect, and does not presuppose intact intuitive social cognition (Livingston and Happé, 2017). We accept this and do not claim that sustained compensation is incompatible with impaired social cognition or executive function; a person may compensate inconsistently and at cost while remaining impaired. The residual point is narrower: where the principal evidence for a diagnosis is a report of successful compensation, and the capacities on which that compensation depends are among those the diagnosis holds to be impaired, the report cannot bear the whole weight of the diagnosis without independent corroboration. In ADHD the point is sharper, because the instruments used to measure camouflaging were derived from autism and have not been validated for ADHD (Adamou, 2026).

None of this implies that clinicians in general use camouflaging as an escape clause, or that adult neurodevelopmental assessment as commonly practised relies on observable traits at a single interview. Responsible assessment integrates developmental history, informant accounts, social communication, cognitive rigidity, sensory profile and functional impairment; it is such assessments that the stronger use of camouflaging is invoked to overturn. Our claim concerns a pattern of inference, visible in contested cases, into which any assessment culture can drift, not the competence of specialists. The consequence for revisability is that a diagnostic culture that permits this pattern of inference cannot revise its judgements, because revision requires the possibility that a diagnosis can be shown to be wrong. Because the construct was developed to explain the missed diagnoses of women and girls, a critique of its stronger use carries an equity risk, addressed in Section 9.

The ethics here are delicate, because the demand to “believe” a masking claim is often framed in the language of epistemic justice. Fricker's (2007) account of testimonial and hermeneutical injustice has been widely, and rightly, applied in disability and psychiatric contexts (Kious et al., 2023), and our own work on second opinions identifies real forms of epistemic injustice in the way experiential knowledge can be discounted (Adamou et al., 2026a). The difficulty is that epistemic justice can be misread as requiring clinicians to treat testimony as beyond correction. To take a patient’s account seriously is to weigh it, allow it to inform the formulation, revise that formulation where appropriate and explain the reasoning transparently; it is not to accept that a self-ascribed diagnosis is immune from assessment. A classification that no testimony could ever challenge would itself fail the principle, because it would remove the patient’s account from the shared process of reasoning, testing and revision.

## 7 Grounds for revising a diagnosis

We have described a culture that, in practice, prevents diagnoses from being revised. Whether this is problematic depends on whether those diagnoses are genuinely stable and firmly established. Here the two conditions must be distinguished, because the case for revisability is substantially stronger for ADHD than for autism, and it rests on different grounds in each. For ADHD the case turns on the instability of the condition; for autism it turns on the reliability of the assessment. Treating “neurodevelopmental diagnosis” as a single object obscures this, and we keep the two apart from here on.

### 7.1 ADHD: the instability of the condition

For ADHD, the evidence that the condition itself changes is now strong. Faraone et al.'s (2006) meta-analysis is often cited for the finding that full-criteria (syndromatic) persistence into adulthood is around 15 per cent by age 25. The same meta-analysis, however, found that when persistence is defined more inclusively, to capture people who retain a subthreshold but impairing set of symptoms, the figure rises to roughly 65 per cent. The honest summary is therefore not that most childhood ADHD disappears, but that a large fraction of those diagnosed as children no longer meet full criteria as adults, while a majority retain some impairment. Agnew-Blais et al. (2016) described both remission and adult-onset presentations in the same cohort, and Sibley et al. (2022), analysing the Multimodal Treatment Study, found that the modal adult course is neither stable persistence nor stable remission but fluctuation: only 9.1 per cent of participants showed sustained remission by the study endpoint, 63.8 per cent had fluctuating periods of remission and recurrence, and most of those who remitted at some point subsequently experienced recurrence. Self-report, on which adult assessment substantially depends, is also more porous than is often acknowledged, as exaggerated or invented symptom presentation is documented among those seeking stimulant access (Fuermaier et al., 2021). The clinical implication is not that ADHD diagnoses are usually wrong but that a diagnosis accurate for a distractible child may, for a substantial minority, cease to describe the adult, and that a fluctuating course is precisely the kind of thing periodic review exists to address and for which the “in partial remission” specifier already provides a form of record. In the terms of Section 3, the ADHD case is principally one of revision in response to change, and only secondarily one of correction.

### 7.2 Autism: the reliability of the assessment

For autism the picture is different, and the claim should be more modest. As a trait, autism is considerably more stable across development than ADHD, and the argument for revisability cannot lean on the condition remitting. Even here, a meaningful minority do not continue to meet the criteria over a long follow-up. Helles et al. (2015), following men diagnosed with Asperger syndrome across two decades, observed the proportion still meeting the original criteria fall from 82 per cent to 44 per cent, although 76 per cent still met criteria for a DSM-IV pervasive developmental disorder at the final follow-up, and prospective work finds diagnoses moving in both directions across development (Elias and Lord, 2022); but the more important consideration for autism concerns not the person but the manner of assessment. In the terms of Section 3, the autism case is principally one of correction.

A growing share of adult diagnostic activity now runs through a rapidly expanded and loosely standardised market. For ADHD, providers offering assessment alone are not required to register with the Care Quality Commission and there is no nationally agreed qualification for those undertaking assessments (Rowland, 2026), and clinicians have described a pattern of rapid, questionnaire-based, remotely delivered assessments in which a positive diagnosis is almost always made (Coghill, 2025). For autism, the United Kingdom independent review found wide variation in conversion rates between providers and raised explicit questions about the consistency, comparability and quality assurance of assessment, particularly under patient-choice pathways (Department of Health and Social Care, 2026); charity investigations have documented conversion rates ranging from near-universal at some services to under a fifth at others (Autistica, 2024). Variation of this kind is a prompt for investigation, not evidence of inappropriate diagnosis. Conversion rates are affected by referral filtering, triage, case mix and service model, as both the review and the charity note; the review states expressly that the variation “does not by itself demonstrate inappropriate diagnosis”. Much assessment has also moved to remote delivery. Whether remote assessment yields less secure autism diagnoses has not been established by diagnostic-accuracy studies; the service-user evidence concerns the acceptability of remote contact rather than its accuracy (Adamou et al., 2021), and the concern rests on the structure of assessment, in which direct observation of communication is a standard component, rather than on any demonstrated effect on validity. We therefore state the position in the only form the evidence supports: where a diagnosis has been made rapidly, remotely, from self-report, by a provider whose conversion rate approaches unity, its security is an open question, and the system currently has no means of answering it. For autism, in short, review is less a matter of tracking change in the person than of quality-assuring an original judgement whose security cannot at present be verified.

Revisability adds something to improved assessment upstream for three reasons: many diagnoses have already been made under the conditions just described, and better future assessment does nothing for them; trait stability is a population generalisation which does not guarantee that any particular label was correctly applied; and some proportion of current diagnoses, which cannot at present be estimated, rest on self-reported presentations that were exaggerated, misremembered or misattributed. For autism, then, revisability is chiefly a quality-assurance capacity directed at diagnoses whose security cannot be verified, not a prediction that autistic people will commonly cease to be autistic. This case is weaker than the case for ADHD; the two conditions appear together here because a single diagnostic culture confers both and the demand-led, identity-conferring and self-reinforcing dynamics operate across both, not because the grounds for revision are equivalent.

## 8 The latent infrastructure of revision

Revision might be thought professionally unprecedented and therefore impractical. It is not: the infrastructure for revising and withdrawing clinical decisions already exists in the closely related domain of medication, and the revision of diagnostic labels at the level of the individual case is established practice in other fields of medicine.

Prescribing is governed by an explicit duty of review. The General Medical Council requires that clinicians have adequate knowledge of a patient’s health before prescribing and, at each review, check that medicines are still needed, effective and tolerated (General Medical Council, 2021). For ADHD, national guidance directs that young people be reassessed at school-leaving age to establish the need for continuing treatment, that adult services carry out a comprehensive assessment after transition, and that medication be reviewed at least once a year to decide with the person whether it should be continued, with clinical need among the matters assessed (National Institute for Health and Care Excellence, 2018, recommendations 1.1.4, 1.1.6 and 1.10.1). None of these recommendations explicitly requires the diagnosis itself to be re-examined: the annual review is a medication review, which presupposes the diagnosis rather than testing it, although a finding that clinical need has lapsed necessarily raises the question. The principle that potent treatment should be actively de-implemented where it no longer serves the patient is institutionalised in STOMP, which enjoins prescribers to stop psychotropic medication lacking current justification in people with intellectual disability and autism (Royal College of Psychiatrists, 2021). Most directly, a recent international consensus on the deprescribing of stimulant medication lists, first among the circumstances in which deprescribing may be appropriate, a diagnosis of ADHD “deemed incorrect upon reevaluation” (Goodman et al., 2026).

The closest precedent, however, is diagnostic de-labelling in other fields of medicine, which shows that revision at the level of the individual case already exists and can be organised. Around 10 per cent of the population of the United States report an allergy to penicillin, yet clinically significant hypersensitivity is uncommon and wanes over time, and structured assessment allows most such labels to be removed (Shenoy et al., 2019). In a prospective Canadian cohort of adults with physician-diagnosed asthma, a current diagnosis could not be established in a third of participants on systematic reassessment, and those whose diagnosis was ruled out were less likely to have undergone objective testing when first diagnosed (Aaron et al., 2017). These programmes treat the label as a claim to be tested rather than a settled fact, distinguish a label that was never warranted from a condition that has remitted, and are justified by the harms of an unwarranted label, including unnecessary treatment and foreclosed options, rather than by cost. They also show that de-labelling requires a low threshold for re-labelling if the condition recurs, which the fluctuating course of ADHD makes essential.

Medicine thus already accepts, and has formal procedures for, the withdrawal of an intervention or a label when it can no longer be clinically justified; removing a diagnosis is likewise a recognised clinical act, not a repudiation of the clinician who originally made it. This both supports and limits the paper’s claim, because the occasions on which a diagnosis could be reconsidered already exist on paper. In practice, however, ADHD reviews appear to concentrate on medication dose and effects and rarely to reopen the diagnosis, which is one of the predictions stated in the Introduction, and no equivalent of de-labelling exists for neurodevelopmental diagnoses. The gap between a review that could, in principle, remove a diagnostic label and one that, in practice, almost never does so is precisely the phenomenon examined in this paper. De-diagnosis is in this sense the counterpart of deprescribing, subject to the qualification in Section 3 that the analogy concerns the current status of a diagnosis and not the historical record. Where diagnosis and medication are closely linked, as in ADHD, continuing stimulant medication entails continuing to affirm the diagnosis that authorises it, and reviewing the medication and reviewing the diagnosis are two aspects of the same responsibility. The absence of a diagnostic counterpart to medication review is not inevitable; it is an oversight, a point at which the profession has yet to extend its established norms from treatment to classification.

## 9 The ethics and politics of diagnostic removal

Everything so far establishes that revising the clinical diagnosis is needed, technically possible, and consistent with what medicine already does. None of it establishes that revision is safe, and the strongest objections to de-diagnosis are not conceptual but ethical and political. We address three in turn: the risk that revision becomes rationing, the possibility that the “stickiness” the argument treats as a defect is in fact a protective feature worth preserving, and the question of who would bear the costs of revision.

The first danger is that a power to remove diagnoses, introduced in the name of accuracy, becomes an instrument of rationing. The conditions are already present: neurodevelopmental services carry waiting lists measured in years, budgets are strained (Smith et al., 2024), and a diagnosis performs a gatekeeper function for treatment, accommodation and, in some cases, welfare support (van der Linden and Schermer, 2024). Where a diagnosis is the gateway to a resource, its removal is a withdrawal of that resource. A programme defended in the language of diagnostic hygiene but driven by the arithmetic of cost would fall hardest on those least equipped to resist it, people without the confidence, literacy or advocacy to contest a clinician’s revised opinion, and the competence paradox sharpens this (Adamou et al., 2026a): a process that reads articulate self-advocacy as evidence against need will remove diagnoses most readily from those who can argue back, or strip them most easily from those who cannot.

The second objection should be stated in its strongest form, because it is the most compelling argument against the entire project: diagnostic stickiness is not a defect but a safeguard. The durability of a diagnosis protects vulnerable people from losing support when an overstretched system finds it convenient to withdraw it, so that a culture in which diagnoses could easily be removed might be more dangerous, not less, than one in which they are difficult to revise; the friction this paper regards as pathological is a hard-won protection.

We think this objection is partly correct. Diagnostic stickiness does protect people, and any revision practice that ignored this protective function would cause harm. Hofmann and Lea's (2022) analysis adds a complication from the other side: the endowment effect, loss aversion and status quo bias in patients, and aversion asymmetry and anticipated regret in clinicians, mean that a system will find withdrawal harder than withholding even where no protective intention is at work, so that stickiness is partly an artefact of bias rather than wholly a safeguard, and the protection it affords is neither designed nor reliable. The objection nonetheless establishes the need for careful design rather than a case against revision itself, for two reasons. First, stickiness protects indiscriminately, preserving securely and insecurely established diagnoses alike. Second, its protective function arises not from the diagnosis itself but from the link between diagnosis and entitlement. If access to support were determined by need rather than by a label, as we argue below, this protection could be provided directly. The objection is therefore decisive against careless revision and against the current fusion of labels with resources, but not against revisability designed with the safeguards that this fusion currently makes necessary.

The Swedish evidence should temper enthusiasm. The qualitative study of the Skåne programme found that participants had often benefited from the support their diagnoses made available, and observed that the very fact that individuals must request, and institutions require, a formal act of removal underscores the enduring power of the classification as both resource and constraint (Lindkvist et al., 2026). The programme also had a feature that limits what can be concluded from it: participants had requested reassessment, and their diagnoses were removed at their request rather than on the service’s initiative. They were a self-selected group especially unlikely to identify with their diagnoses, and the programme shows that patient-initiated revision is possible without showing how the wider diagnosed population would respond to routine review. De-diagnosis initiated by a patient and de-diagnosis initiated by a service are fundamentally different practices: the first is an exercise of autonomy that services should be able to respect; the second places considerable power over identity and access to support in the hands of the service and must meet a much higher ethical standard.

The third objection concerns who would bear the costs of revision, which earlier formulations of this argument did not adequately address. The camouflaging construct was developed to explain the missed diagnoses of women and girls, and masking is arguably better understood as a response to stigma than as a gendered trait (Pearson and Rose, 2021); a revision practice that treated reliance on masking as a marker of an insecure diagnosis would fall disproportionately on late-diagnosed women, the group whose under-recognition the construct was introduced to correct. Diagnoses obtained through private and patient-choice routes are unlikely to be evenly distributed by income or region, and the independent review has identified the contribution of these routes, and its variation by region and social group, as an unresolved question (Department of Health and Social Care, 2026); a revision practice directed at those routes would have a social gradient. Recorded ADHD diagnosis and prescribing in UK primary care are, by contrast, associated with deprivation (McKechnie et al., 2023), so that a practice which reviewed NHS caseloads would have a different gradient again. Any revision practice will have distributional consequences, which must be anticipated and monitored rather than discovered afterwards; this is the basis of the equity requirement in Section 10.

Beneath these questions lies the issue of professional power that sociologists of the professions have examined for decades (Freidson, 1970; Jutel, 2011). The authority to remove a diagnosis extends the authority to make one and raises the same concerns about medical dominance, at the point where the clinical relationship may be most strained. Handled badly, de-diagnosis would confirm the darkest criticism of psychiatry, that the profession assigns and withdraws identities at will and in its own interests; handled well, it could show a profession willing to correct its own record in the patient’s interest rather than the system’s.

A defensible practice must also review a diagnosis with the patient rather than act upon it, giving serious weight to their account, explaining the reasons for any change, and accepting that the patient may be right and the original diagnosis wrong, without placing a self-ascribed diagnosis beyond assessment. A programme of diagnostic revision is legitimate only if it can show, in each case, that it serves the patient’s epistemic and practical interests rather than the system’s financial interests; when it cannot, it is rationing presented as diagnostic accuracy.

## 10 Design requirements for a defensible practice of revision

Recognising that diagnoses can be revised is not the same as creating a formal process for revising them. The requirements below are conditional: neither a service specification nor a recommendation to create a service, but the minimum requirements any defensible practice of diagnostic revision must meet, set out partly to show how difficult they would be to satisfy. If they cannot be met, revision should not proceed.

First, the trigger for review. A review must begin with epistemic doubt, not financial pressure. There must be a specific, documented reason to believe that the diagnosis no longer applies to the person, not a target to reduce prescribing or to discharge a group of patients. Who initiates the review, and on what grounds, marks the boundary between quality assurance and cost control.

Second, the distinction between routes to review. As the Swedish programme shows, de-diagnosis requested by a patient and de-diagnosis initiated by a service across a caseload must meet different standards (Lindkvist et al., 2026): exercising personal autonomy should be easy, and exercising institutional power over patients should be difficult. The same principle applies in the other direction: a patient’s request to have a non-diagnosis reconsidered should be as accessible as a request to have a diagnosis reconsidered, and a revisable culture is one in which both requests are ordinary.

Third, independent scrutiny. The second-opinion data show that a patient’s articulate self-advocacy can easily be interpreted as evidence that they do not need support. Any contested removal should therefore be reviewed by someone other than the clinician proposing it, and the patient should have a clear right of appeal (Adamou et al., 2026a).

Fourth, a firewall between diagnosis and resources. Any effects on funding or eligibility that might follow from a diagnostic change should be separated from the diagnostic decision itself, so that clinical judgement is neither influenced by financial pressure nor reasonably perceived to be so.

Fifth, and least often discussed but the most important, a diagnosis must be separated from the support attached to it; this requirement also answers the objection that stickiness provides necessary protection. Losing a diagnostic label should not automatically cost a person workplace adjustments, therapy or access to services. Much of the fear surrounding de-diagnosis arises from the link between diagnosis and entitlement, which has given labels an administrative function they were never designed to perform; if support were determined by need rather than by the continued presence of a label, diagnoses could be revised without depriving people of necessary support. This requirement is easier to state than to meet, and the education literature, where the debate is mature, shows why. Norwich (2008), drawing on Minow (1990), described the “dilemma of difference”: identifying children as different in order to provide for their needs risks stigmatising them, while declining to identify them risks leaving needs unmet. Categorical labels in special education secure resources but carry stigma and lowered expectations, and their usefulness as a basis for allocating provision is contested (Lauchlan and Boyle, 2007). English law already defines special educational needs functionally, by reference to a learning difficulty or disability that calls for special educational provision, rather than by diagnosis (Children and Families Act, 2014, s. 20), yet in practice a diagnosis remains the currency through which needs are recognised, exactly as with the Equality Act. Needs-led provision therefore requires more than a change of rule: it requires assessment capacity able to establish need without a label, and institutions willing to act on such assessments. Two consequences should be stated frankly. If support were determined by need, the demand for diagnosis would itself change, because part of what drives demand is the entitlement the label carries; the causal account in Sections 4 and 5 is, to that extent, an account of the present administrative arrangement rather than of any necessary feature of diagnosis. And if this requirement cannot be met, service-initiated revision should not proceed, because the protective function of stickiness would then have no substitute.

Sixth, differentiation between the two conditions. Adults seek ADHD and autism assessments for different reasons, and the justification for revision differs, arising from change over time in ADHD and from uncertainty about the original assessment in autism. A review that treats an ADHD diagnosis linked to stimulant medication as equivalent to an autism diagnosis with no associated medication will misunderstand both.

Seventh, an explicit equity criterion. Because any revision practice has distributional consequences, its effects must be monitored by sex, age at diagnosis, route to diagnosis, deprivation and ethnicity, and a practice that removed diagnoses disproportionately from any group, or that treated reliance on masking as in itself a marker of an insecure diagnosis, should be halted pending independent review. Equity is not an addition to the other requirements but a test of whether they are being met.

Meeting all seven requirements would be difficult. A programme that cannot meet them should not proceed, because it would amount to rationing under the language of diagnostic accuracy; the requirements establish not only how a diagnostic review should be designed but when it should be refused.

## 11 Discussion

### 11.1 Contribution to the current debate

This analysis contributes to three related bodies of literature.

The first contribution is to the sociology of diagnosis. Blaxter, Brown and Jutel established that diagnosis is a consequential act of classification, Freidson placed that act at the heart of professional power (Blaxter, 1978; Brown, 1995; Freidson, 1970; Jutel, 2009), labelling theory explained why labels persist, and the overdiagnosis literature explained how unwarranted diagnoses arise. Far less attention has been paid to the revision of an individual diagnosis within an ongoing clinical relationship, especially in adult neurodevelopmental psychiatry; Hofmann and Lea (2022) have examined the ethics of withdrawing unwarranted diagnoses, and this paper extends their analysis by specifying the mechanisms that make withdrawal unusually hard in this field and by distinguishing the forms that revision can take. The concept of revisability brings into view a capacity that falls between a sociology concerned with the assignment of diagnoses and a demedicalisation literature focused on categories rather than individual cases, and that must be understood, like conferral, as an exercise of professional power.

The second contribution is to the debate on medicalisation, which changes once medicalisation is recognised as increasingly co-produced, with patients among its principal authors (Correia, 2017). When the demand for diagnosis comes partly from patients, restricting the power of providers is no longer a sufficient response; another safeguard is needed, a legitimate way to leave behind a diagnosis that no longer fits, because medicalisation sought by the patient carries the distinctive risk that, once achieved, it may be difficult to reverse.

The third contribution is to the philosophy of human kinds. Hacking’s account of interactive kinds and looping effects has been widely used to explain how classifications shape the people to whom they are applied (Hacking, 1999, 2007). Here it serves a more specific and deliberately bounded purpose: to explain why medicalisation sought by patients can become self-reinforcing, and why the forces that expand a diagnostic category can also make individual diagnoses harder to revise, since a diagnosis may shape how a person understands themselves, so that its removal feels less like a correction than the loss of part of an identity. The account is not used to establish that the category has lost validity, which it cannot do.

The paper’s overall claim is deliberately limited. It does not propose de-diagnosis as a service, nor does it endorse or reject the Swedish programme, which is used only to clarify the questions at stake. It does not suggest that adult neurodevelopmental diagnoses are usually wrong, that the meaning people find in them is mistaken, or that the neurodiversity movement is responsible for the problem. Its claim is that revisability is a feature a diagnostic culture may or may not have, that adult neurodevelopmental psychiatry currently lacks it although medication review and de-labelling practice elsewhere in medicine provide some of the necessary foundations, and that this absence is a shortcoming that can, in principle, be addressed.

### 11.2 Limitations

The empirical illustrations are drawn from the first author’s own studies and from corpora generated within NHS services of the kind he leads; they are selected for contestation and cannot support claims about the general population of people assessed. The analysis is situated in the United Kingdom, and the administrative fusion of diagnosis and entitlement that it describes may take different forms in other systems. The claim that demand is now substantially patient-led is directional and unquantified. The case for revisability in autism rests on the security of assessment, which the available evidence can question but not measure, and it is accordingly weaker than the case for ADHD. Finally, the argument is conceptual, and although it generates testable predictions, none has yet been tested.

### 11.3 Concluding remarks

Recorded diagnoses of adult ADHD and autism, together with the demand for assessment, appear to have risen more quickly than any plausible change in underlying prevalence (Department of Health and Social Care, 2026; McKechnie et al., 2023; Russell et al., 2022). The sociology of diagnosis has examined the assignment of diagnoses in detail (Jutel, 2009) while giving much less attention to their removal in individual cases, and clinical practice reflects the same imbalance: there is no routine process for reconsidering a diagnosis, even where an occasion formally exists, as it does in the annual ADHD medication review (National Institute for Health and Care Excellence, 2018). Diagnosis, understood as defeasible inference, is revisable by its nature; the diagnostic culture has simply not built the means to exercise that property in one direction.

Three developments explain how this omission has become a practical inability to revise diagnoses. First, demand for diagnosis now comes in substantial part, though in a proportion that cannot be quantified, from patients themselves, in interaction with clinicians, advocacy, markets and administrative systems, so that limiting the power of medical providers is no longer a sufficient response to medicalisation. Second, diagnosis has become a source of identity: through the looping effect, diagnostic categories shape the people they classify, who in turn shape and strengthen the categories (Hacking, 1999, 2007), and the process which expands a diagnosis can also make it resistant to revision. Third, the expansive use of the camouflaging construct can make diagnoses difficult to disconfirm, because when an absence of expected features is interpreted as evidence of concealment, almost any observation can be made consistent with the diagnosis; this criticism applies to an unrestricted use of the construct, not to its legitimate and bounded application, nor to the competence of clinicians who assess responsibly.

The case for revision differs between ADHD and autism. ADHD symptoms often fluctuate, and a substantial minority of people diagnosed in childhood no longer meet full criteria in adulthood (Biederman et al., 2010; Sibley et al., 2022); autism is generally stable as a trait (Whitehouse et al., 2011), but many adult assessments now take place in a market whose consistency and reliability have been questioned. Some diagnoses may cease to fit because the person has changed, while others may never have been securely established, and the typology in Section 3 is intended to supply the different responses these situations require. The profession already has a model in its duty to review medication, and a closer one in the de-labelling of penicillin allergy and the reassessment of adult asthma. The remaining obstacles are conceptual, where broad interpretations of camouflaging protect a diagnosis from disconfirmation, and ethical and political, because removing a diagnosis may restrict access to services, may fall unequally on particular groups, and may unsettle an identity the diagnosis helped to shape.

Any practice of revisability must therefore meet demanding conditions: reviews that begin with genuine doubt rather than pressure to reduce costs; a distinction between patient-requested reassessment and review imposed by a service, with the reconsideration of non-diagnoses as accessible as the reconsideration of diagnoses; independent scrutiny of disputed decisions; clinical judgement protected from financial influence; continued support that depends on need rather than on the persistence of a label; monitoring of distributional effects; and the treatment of ADHD and autism as distinct clinical and social objects. If these conditions cannot be met, a diagnostic revision system should not be introduced.

To take diagnosis seriously as a form of knowledge is to accept that it can be mistaken. It also requires the ability to acknowledge and correct a mistake without depriving the patient of the support the diagnosis has made possible. The capacity to address such diagnostic errors effectively remains limited, and this absence should concern us.

## Data Availability

The original contributions presented in the study are included in the article/supplementary material, further inquiries can be directed to the corresponding author.
